# Olympic combat sports and mental health in children and adolescents with disability: a systematic review of controlled trials

**DOI:** 10.3389/fpsyg.2025.1567978

**Published:** 2025-05-01

**Authors:** Youngjun Lee, Flavia Guidotti, Laura Capranica, Caterina Pesce, Valentin Benzing, Janet Hauck, Simone Ciaccioni

**Affiliations:** ^1^Department of Kinesiology, Michigan State University, East Lansing, MI, United States; ^2^Department of Movement, Human and Health Sciences, University of Rome “Foro Italico”, Rome, Italy; ^3^Department of Human Sciences and Promotion of the Quality of Life, San Raffaele Roma Open University, Rome, Italy; ^4^Institute of Sport Science, University of Bern, Bern, Switzerland; ^5^Department of Education and Sport Sciences, Pegaso Telematic University, Naples, Italy

**Keywords:** Olympic combat sports, mental health, children, adolescents, disability

## Abstract

**Introduction:**

Children and adolescents with disabilities face increased mental health challenges and lack of access to exercise. Olympic combat sports (OCS) such as judo, taekwondo, and others might encourage social engagement, self-control, and resilience. However, not much is known about their mental health impact on this population.

**Methods:**

Following PRISMA-P protocols (PROSPERO registration: CRD42023452489), we searched seven databases for randomized controlled trials or non-randomized trials that evaluated the impact of OCS on mental health in children and adolescents (5–18 years) with developmental or physical disabilities. The key findings fell into 11 domains across mental illness attitudes, social skills, and mental health literacy. We also extracted individual (e.g., age) and social (e.g., family participation) moderating factors. Rob 2.0 (randomized trials) and ROBINS-I (non-randomized trials) were used to measure the risk of bias.

**Results:**

Twelve studies (seven randomized, five controlled trials) conducted during 1975–2022, encompassing 436 participants (11.4 ± 2.8 years), were included. There were significant improvements (*p* < 0.05) in stereotypy, communication, social–emotional functioning, and executive function, with occasional improvements in self-esteem and stress management. Several experiments reported rapid hormonal changes (e.g., cortisol) immediately after OCS, particularly among adolescents. Family involvement and age emerged as potential moderators, with older children and adolescents with engaged carers likely to benefit even more.

**Discussions:**

Despite different study protocols, outcomes, and risk-of-bias thresholds, OCS interventions overwhelmingly seem to enhance mental health in children and adolescents with disabilities. More substantial, longer-term trials would be required to validate these findings, explain the processes, and evaluate safety. Programs based on OCS that address disabilities could provide broad pathways to physical activity and psychological development as part of a whole-person developmental model.

**Conclusion:**

This review indicates that OCS interventions have the potential to improve mental health outcomes for children and adolescents with disabilities by increasing social skills and executive functioning while better regulating stress. The diversity of samples and inadequate study designs necessitate additional high-quality research.

**Systematic Review Registration:**

https://www.crd.york.ac.uk/PROSPERO/view/CRD42023452489, CRD42023452489.

## 1 Introduction

For athletes, the Olympic Games celebrate solidarity, excellence, and human achievement (Girginov and Parry, 2005). While elite athletes often represent the pinnacle of physical performance, Olympic sports also hold broader relevance for the general population, particularly youth (Bauman et al., 2021; Craig and Bauman, 2014). Olympic combat sports (OCS), such as boxing, fencing, judo, karate, taekwondo, and wrestling, are particularly well-suited for youth development due to their unique combination of physical exertion, structured routines, and cognitive-social engagement. Although commonly viewed through the lens of competition, OCS provides developmental opportunities through its repetitive structure, rule-based environment, and emphasis on respect and self-control (Fukuda and La Monica, 2017). OCS also require rigorous cardiovascular conditioning since they involve repeated bursts of intense physical activity. As a result, participants in these sports generally have a high level of aerobic fitness. This excellent fitness translates into improved cardiovascular well-being, prevention of cardiovascular diseases, and increased endurance (Fukuda and La Monica, 2017; Valdés-Badilla et al., 2022). Effective engagement in OCS fosters the development of functional strength muscular power, and hypertrophy through a combination of resistance exercises, bodyweight workouts, and sport-specific movements tailored to the sport’s unique demands (da Silva Santos and Franchini, 2021; Ciaccioni et al., 2019; Miller et al., 2022). Importantly, these characteristics may be relevant to children and adolescents with disabilities, who often benefit from structured environments and movement-based programs that support emotional regulation, physical literacy, and opportunities for social engagement (Ash et al., 2017).

The biopsychosocial model serves as the basis for conceptualizing “disability” in this review, which follows the World Health Organization’s International Classification of Functioning, Disability and Health (ICF) (Rosenbaum and Stewart, 2004). According to this model, disability emerges from the interaction between personal health conditions such as developmental or physical impairments and contextual elements, including environmental or social obstacles that impact individuals’ daily life participation and functioning. This broad framework enables examining multiple types of disabilities, such as developmental, intellectual, physical, and sensory impairments, which appeared in the selected review studies. Children and adolescents with such conditions often face elevated risks of anxiety, depression, and social isolation (Emerson, 2003; Buckley et al., 2020; Pinquart and Shen, 2011)—making early mental health support through accessible, engaging interventions critically important. With an emphasis on holistic development, OCS provides structured, active exercise with a wide range of benefits, especially for youth with disabilities, who can nurture a sense of inclusion and belonging while fostering life skills to overcome unfair barriers (Antunes et al., 2018; Boguszewski and Torzewska, 2011; Kadri et al., 2019; Milligan et al., 2015; Carroll and Allen, 2021). This review does not focus on athletic achievements but examines how OCS can improve mental health outcomes for children and adolescents with disabilities. Through participation in OCS, children with disabilities experience structured environments that support psychological resilience building, emotional regulation skills development, and a sense of belonging, which are critical throughout their formative developmental years (Lyu and Gill, 2011; Hutzler and Korsensky, 2010; Shields and Synnot, 2016; Fraser-Thomas et al., 2005).

Following the COVID-19 pandemic, concerns regarding youth mental health have grown, with reporting increased anxiety, depression, and stress levels. A cross-sectional study of almost 584 youth found that 40.4% had psychological difficulties, and 14.4% developed symptoms of post-traumatic stress disorder (PTSD) 2 weeks into the pandemic, underscoring the early prevalence of mental health problems among this demographic (Liang et al., 2020). These are especially prevalent in children and adolescents with disabilities who are predisposed to physical inactivity (Patel, 2020; Leung et al., 2023). Lockdowns and social distancing further limited access to supportive networks, adaptive physical education, and inclusive sports environments, which further eroded their mental health and physical activity levels. This waning of habitual activity and socialization has revealed the need for systematic, convenient forms of exercise that encourage psychological resilience, social connectedness, and whole-person well-being. In this respect, OCS might present an inclusive platform – combining the physical, cognitive, and social spheres in ways that allow young people with disabilities to make up ground lost in motor skill development and protect mental health. More than 6.5 million American children participate in martial arts, including OCS, such as judo and taekwondo, alongside karate (Demorest et al., 2016). In Australia, children participation in martial arts for males peaked at 9–11 years of age, and 1.2% of the 15 + population engages in martial arts, with 85% of participation being organized and total annual spending on martial arts reaching $87 million (Australia, 2019). However, existing epidemiologic data lacks participation rates data for children and adolescents with disabilities, demonstrating a deficiency in both academic studies and inclusive program development.

Despite the above claims about the overarching potential benefits of OCS for persons with disability (Antunes et al., 2018; Boguszewski and Torzewska, 2011) and specifically during development (Kadri et al., 2019; Milligan et al., 2015), research into the relationship between OCS and the mental health of children and adolescents with disabilities remains largely unexplored in the literature. The current evidence base is further limited by small sample sizes, heterogeneous methodologies, and a lack of longitudinal data—underscoring the need for a systematic synthesis of existing trials. This review evaluates mental health outcomes—such as communication, social competence, executive function, self-esteem, stress regulation, as well as reductions in stereotypy, aggression, and improvements in mood and adaptability—that are especially important for children and adolescents with disabilities (Buckley et al., 2020; Emerson and Hatton, 2007). This review will give a starting point for future research to help fill the gaps in what we know about the association between OCS and mental health in this population. This review’s primary aim is to locate, analyze, and summarize published studies that investigated whether involvement in OCS influences the mental health of children and adolescents with disabilities. The objectives for this review, including subgroup analyses from sections 2.1 and 2.2, were established beforehand by the registered PROSPERO protocol number CRD42023452489. The framework directed the review process while maintaining analytical consistency throughout. The research team made minimal adjustments, including adding executive function as an outcome category based on repeated patterns in multiple studies. Subgroup syntheses will be performed to identify whether different dimensions of the overall construct of mental health are differentially influenced by OCS practice and whether individual factors (e.g., age) and social proximal factors (e.g., family) may play a role in the relation between OCS and mental health in children and adolescents with disabilities.

Therefore, the primary purpose of this review is twofold: First, conduct a systematic review of empirical studies examining whether OCS participation influences the mental health of children and adolescents with disabilities. Second, more specifically, identifying (2.1) how OCS interventions affect mental health across 11 core outcome domains (grouped under three significant constructs) and (2.2) how individual factors (e.g., age) and social proximal factors (e.g., family involvement) might moderate or shape these mental health outcomes.

## 2 Methods

This systematic review followed the Preferred Reporting Items for Systematic Reviews and Meta-Analysis Protocols (PRISMA-P) guidelines (Supplementary Material 1) (Moher et al., 2015). The full study protocol and search details were prospectively registered in PROSPERO (CRD42023452489) and accepted (Lee et al., 2025).

### 2.1 Population

Inclusion criteria included peer-reviewed studies, such as randomized controlled trials (RCTs) and clinical trials (CTs), published in international journals focused on OCS programs explicitly aimed at children and adolescents (ages 5–18) with developmental, emotional, intellectual, physical, and sensory disabilities across developmental areas (emotional, intellectual, physical, and sensory); and interventions including treatment for both acute and chronic disability in children and adolescents. Exclusion criteria were qualitative research, reviews, meta-analyses, reports, protocols, letters, editorials, working papers, conference proceedings, theses/dissertations, and gray literature; studies in which children or adolescents had persistent diseases that limited their physical activity – even those being treated at any stage; studies with participants suffering from cancer or anterior cruciate ligament injuries and research from clinical trial settings; studies reporting information on an age group outside the defined range unless providing specific information about a subgroup within our defined range. At the outset, our research included multiple study designs to collect evidence. However, our focus sharpened to only experimental and quasi-experimental methods to improve our ability to make causal inferences. Consequently, we excluded observational study designs, including cross-sectional case–control and cohort studies. Some observational studies were identified during the initial search but were excluded during the full-text screening phase.

### 2.2 Outcome

**Figure 1:**
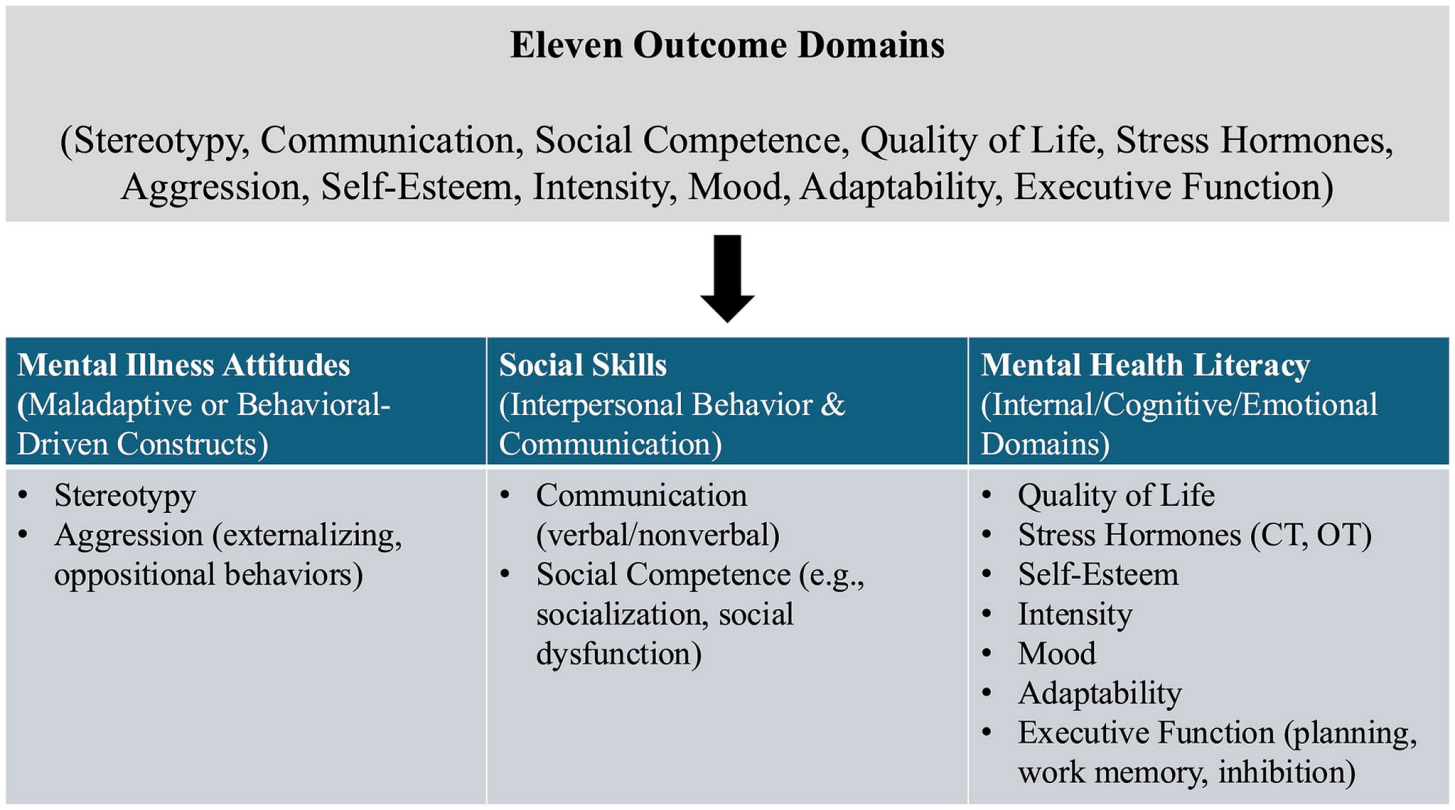
Flowchart mapping the eleven outcome domains onto three overarching constructs. CT, cortisol; OT, oxytocin.

First, our protocol measured eleven outcome domains: stereotypy, communication, social competence, quality of life, stress hormones, aggression, self-esteem, intensity, mood, adaptability, and executive function. We positioned these results within three broad constructs, (Girginov and Parry, 2005) mental health literacy, (Bauman et al., 2021) mental illness attitudes, and (Craig and Bauman, 2014) social skills, to provide conceptual precision as well as congruence with the various outcome measures documented in the included studies (Fusar-Poli et al., 2020). Communication and social competence, for example, naturally fell into the category of social skills. At the same time, stereotypy and aggression remained closer to attitudes toward mental illness, especially in the context of developmental or intellectual disabilities. Self-esteem, mood, intensity, executive function, mental health literacy (e.g., self-awareness and control), and attitudes toward mental illness (e.g., externalizing vs. internalizing behavior). We decided to include executive function because it was common in the OCS literature but not in our original seven domains. Above all, most experiments rated planning, working memory, or inhibitory control in the larger field of mental health, which we needed to acknowledge explicitly in our integrated model. We also swapped out “physiological responses” for “stress hormone” to keep the terms consistent and separate them from purely behavioral effects. Third, stereotypy is a common behaviorally driven attribute in developmental and intellectual disabilities, although rarely examined in general-population mental health studies. In this way, our new 11-domain grid is both respectful of the original protocol and responsive to the heterogeneous findings resulting from studies of children and adolescents with disabilities engaged in OCS programs. We also recorded information on participant age and family-related factors (e.g., presence of parents during training), hypothesizing that these might moderate the relationship between OCS interventions and mental health outcomes (Figure 1).

### 2.3 Interventions and study design

Seven electronic databases (Scopus, ERIC, PsycINFO, PubMed, Cochrane Library, Web of Science, and SPORTDiscus) were considered to search for RCTs and CTs studies focused on OCS-based programs for children and adolescents with disabilities. The literature search across the seven databases was conducted between July 23 and July 26, 2023. The search strategy targeted four significant variables: (1) OCS, (2) mental health, (3) the demographic target group of children and adolescents with disabilities, and (4) study designs and associated terminologies. The review focused solely on Olympic combat sports (OCS)—boxing, judo, karate, taekwondo, wrestling, and fencing—which have received official recognition from the International Olympic Committee (IOC). This selection ensured definitional clarity, consistent intervention typologies, and alignment with the original PROSPERO protocol. Given the vast scope of martial arts and combat sports, narrowing the focus to OCS allowed for a more structured analysis based on standardized rules, global recognition, and high-performance contexts. While promising evidence exists for other combat sports, such as Brazilian jiu-jitsu (Bueno et al., 2023), capoeira (Fernandes et al., 2022), and mixed martial arts (MMA) (Phung and Goldberg, 2021), these were excluded due to their non-OCS status. Moreover, Olympic combat sports receive significant research, funding, and policy attention, facilitating comparability, access to quality data, and scientific investigation while avoiding an overly broad scope. Literature not in English was included where a translation in English existed or could be obtained with the help of the review team. Additionally, we used a snowballing approach, screening all articles included in our study through reference lists alongside database searches. Five additional studies were identified for a full-text review from this process, but they failed to meet our inclusion criteria, which led to their exclusion from the final synthesis. Our review only included studies that were published starting in 1975. Our preliminary scoping review determined that 1975 was the publication year of the earliest acceptable study, satisfying all inclusion criteria, which led to our decision. In addition, the mid-1970s saw the development of organized physical activity programs alongside scientifically rigorous methods within disability research (Rimmer, 1999; Sherrill, 1998). Also, studies published from 1975 maintain methodological consistency and preserve historical relevance.

### 2.4 Data extraction and quality appraisal

Data extraction was achieved by creating a data extraction template in Covidence and running a pilot trial before starting the data extraction process. Two independent reviewers screened each study. If something did not exist or needed clarification, we consulted the authors of the relevant studies. If no response came before the data extraction or the reporting was completed, the study was not included in the review. Once independent data was extracted, the two reviewers participated in a consensus process to resolve differences and verify the extracted data’s validity. Whenever disagreements arose between reviewers during the screening and data extraction processes, a consensus was achieved through discussion. When reviewers disagreed, we sought help from an independent third-party researcher. The information obtained contained the following elements:

- Study and Intervention Details: These included the study design, a brief overview, information on the intervention’s design and contents, descriptions of the control group’s physical or non-physical activities, and the study location.
- Participant Information: This section captured the sample size, participants’ ages (including age distribution by gender), gender distribution (broken down by percentage), and type of disability.
- Outcome Measures and Modifiable Factors: This component encompassed various outcome measures and modifiable factors.
- Timeframes: Information regarding the duration of the intervention (in weeks), the location of the intervention (country), the number of measurement time points, and the duration of follow-up (in weeks).
- Results Data: This section included outcome data (mean values and measures of variance) and data related to modifiable determinants (mean values and measures of variance).

**Figure 2:**
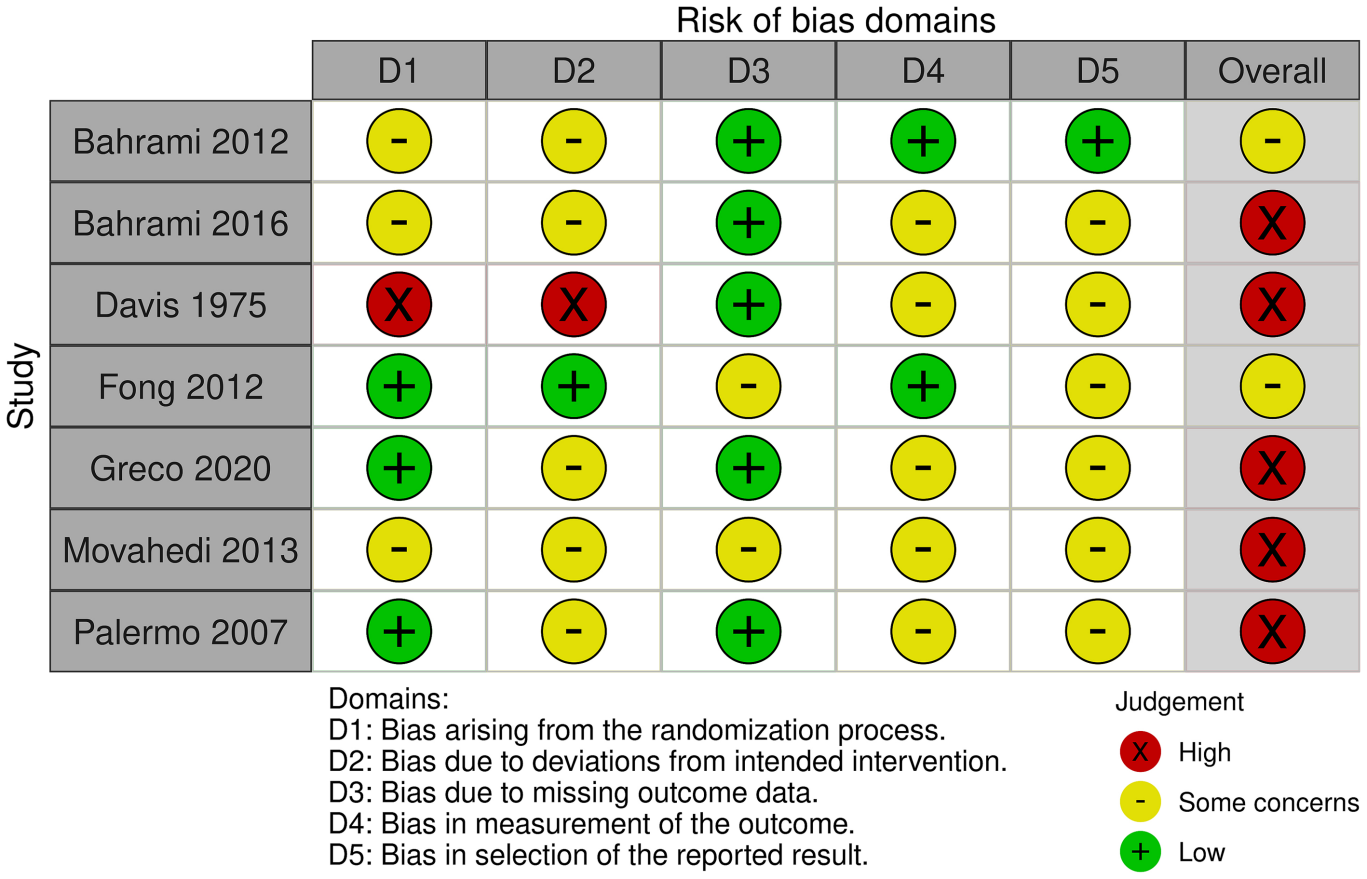
Risk of bias for the selected RCT.

**Figure 3:**
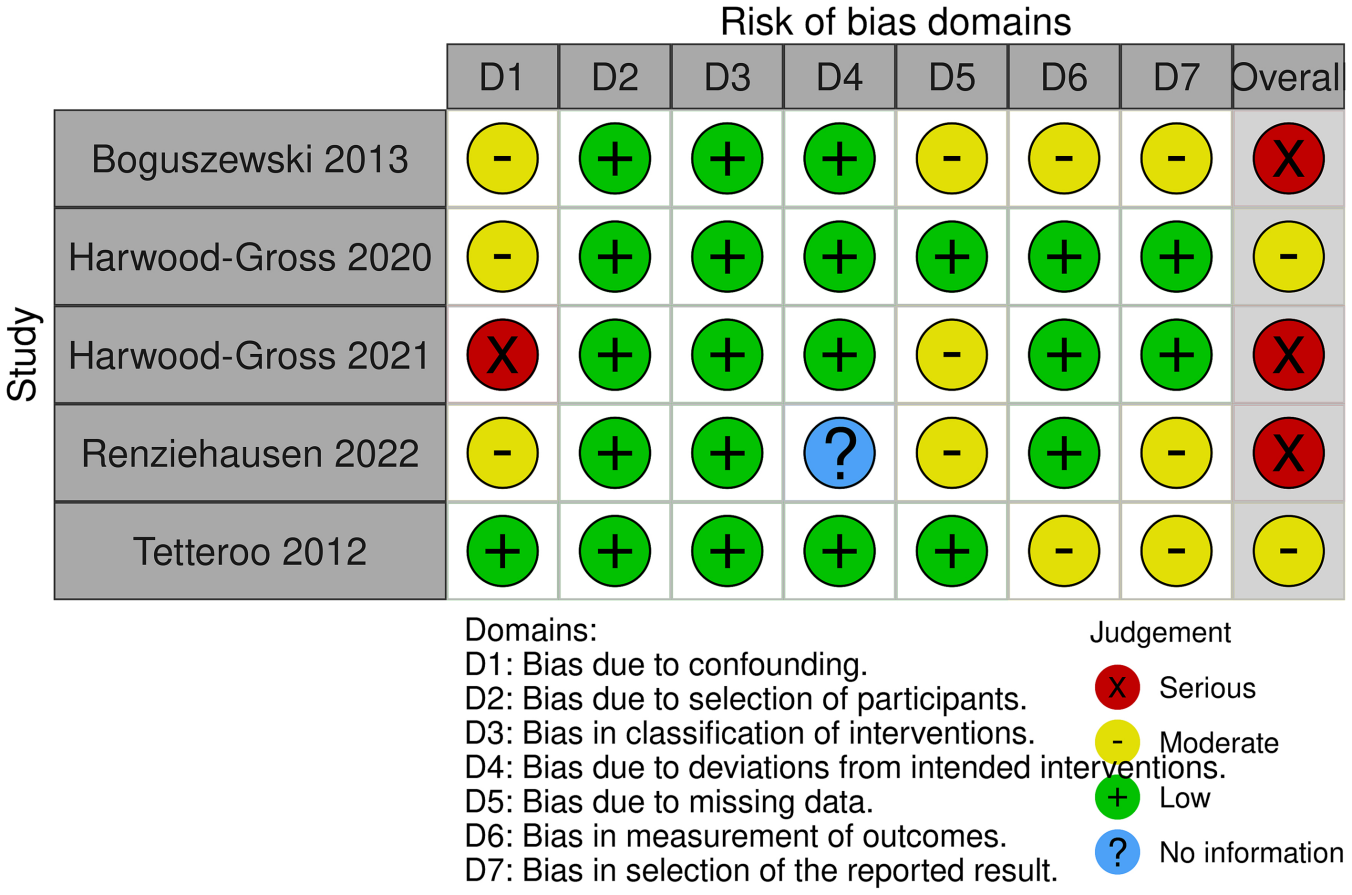
Risk of bias for the selected CT.

A risk of bias analysis was performed for all study designs to assess the included studies’ methodological rigor and internal validity. Appropriate assessment tools were used for each study type, and according to the Cochrane Collaboration, RCTs adapted the Rob 2.0 tool (Higgins et al., 2011). This instrument encompasses the following areas: bias from randomization, bias due to non-consistent interventions, bias due to incomplete outcome information, bias in outcome measurement, and bias in the reported outcome selection. In the case of non-RCTs, the risk of bias was determined using a modified version of the ROBINS-I (Risk Of Bias In Non-randomized Studies– of Interventions) measure (Sterne et al., 2016). The tool assesses the following areas: confounding bias, participant selection bias, intervention classification bias, deviations from planned exposures or interventions, missing data bias, outcome measurement bias, and outcome selection bias of reported results. Two independent reviewers (who extracted data themselves) conducted risk of bias assessments. For this survey, they used forms made in Covidence, with templates for specific risk of bias calculators. After each reviewer independently extracted data, they agreed to reconcile any disagreements and ensure their assessments were correct. Most RCTs were considered to have either “Low” or “Some concerns” in areas including randomization, outcome measurement, and reporting. However, two RCTs (Davis and Byrd, 1975; Movahedi et al., 2013) had a “High” overall risk, primarily due to randomization errors or deviations from the intervention. Of the non-randomized trials, the combined scores ranged from “Moderate” to “Serious,” typically due to confounding variables or not properly recording outcome data. None of the studies included received a “Critical” rating that would discredit them. Despite these disparate sources of bias, most studies were of satisfactory quality, and the aggregated findings must be viewed cautiously. Nevertheless, they must provide valuable guidance as to how OCS interventions might influence the mental health of children and adolescents with disabilities (Figures 2, 3).

### 2.5 Method of synthesis

Because of significant variations in the measurement tools, intervention forms, and reported effects among the studies included, we ran a narrative SWiM according to the recommendations of Campbell et al. (2020). We grouped the studies according to (a) intervention time (acute vs. chronic), (b) participant characteristics (e.g., disability type), and (c) reported outcome domains (e.g., emotional-behavioral, cognitive/executive, physiological). Because the experiments used different outcome measures (e.g., rating scales, hormone testing), we rely almost entirely on the authors’ reported effect sizes, mean, and *p*-values without statistical transformation. Where effect sizes were unavailable, we calculated the direction and magnitude of effects qualitatively based on significance (*p* < 0.05) or descriptive trends. So, we summed up outcomes by defining effect directions for each outcome domain. We focused on consistent outcomes across studies (e.g., stereotypy, executive function). To manage heterogeneity, we looked at differences in participant age, intervention frequency/length, and disability subtype in interpretative interpretations. The substantial heterogeneity observed in the small sample size resulted in us not performing formal statistical tests such as the Q-statistic. In addition, substantial differences among studies regarding outcome domains, measurement tools, and varying reporting formats prevented us from performing a meta-analysis. The statistical reporting in multiple studies did not include standard metrics such as means, standard deviations, or effect sizes. At the same time, the overlapping outcome constructs did not allow for effective aggregation of effect sizes. We used a parsimonious approach to evaluate evidence confidence, examining whether each outcome was accompanied by at least one positive effect and whether that effect was replicated or refuted by further studies. We recognize that our replication method lacks scientific rigor and excludes statistical strength evaluations and bias risk assessments. The formal GRADE assessment proved unattainable because of differing outcome scales, unstandardized reporting practices, and limited participant numbers, suggesting that future reviews need more powerful grading methods. The output describes the direction, meaning, and similarity of outcomes domain by outcome domain. We also recognize some shortcomings of this synthesis, such as a low sample size, varying study design, and use of reported *p*-values rather than effect-size metrics. We then viewed our results using the Consolidated Framework for Implementation Research (CFIR), which includes five domains: the type of intervention, contexts outside and within it, individuals, and implementation (Damschroder et al., 2022). Through CFIR, we can situate the OCS interventions within more extensive real-world settings and identify factors (e.g., setting and stakeholder involvement) that could influence outcomes. Due to significant methodological differences in study design and other factors, we avoided formal statistical heterogeneity testing, such as the Q-statistic, because it was considered unsuitable. We utilized structured narrative synthesis, SWiM, to organize studies according to outcome domains and intervention features before interpreting effect patterns through descriptive comparisons. This blended SWiM–CFIR approach offers an interpretive framework that addresses intervention diversity while partially compensating for the lack of statistical synthesis.

## 3 Results

**Figure 4:**
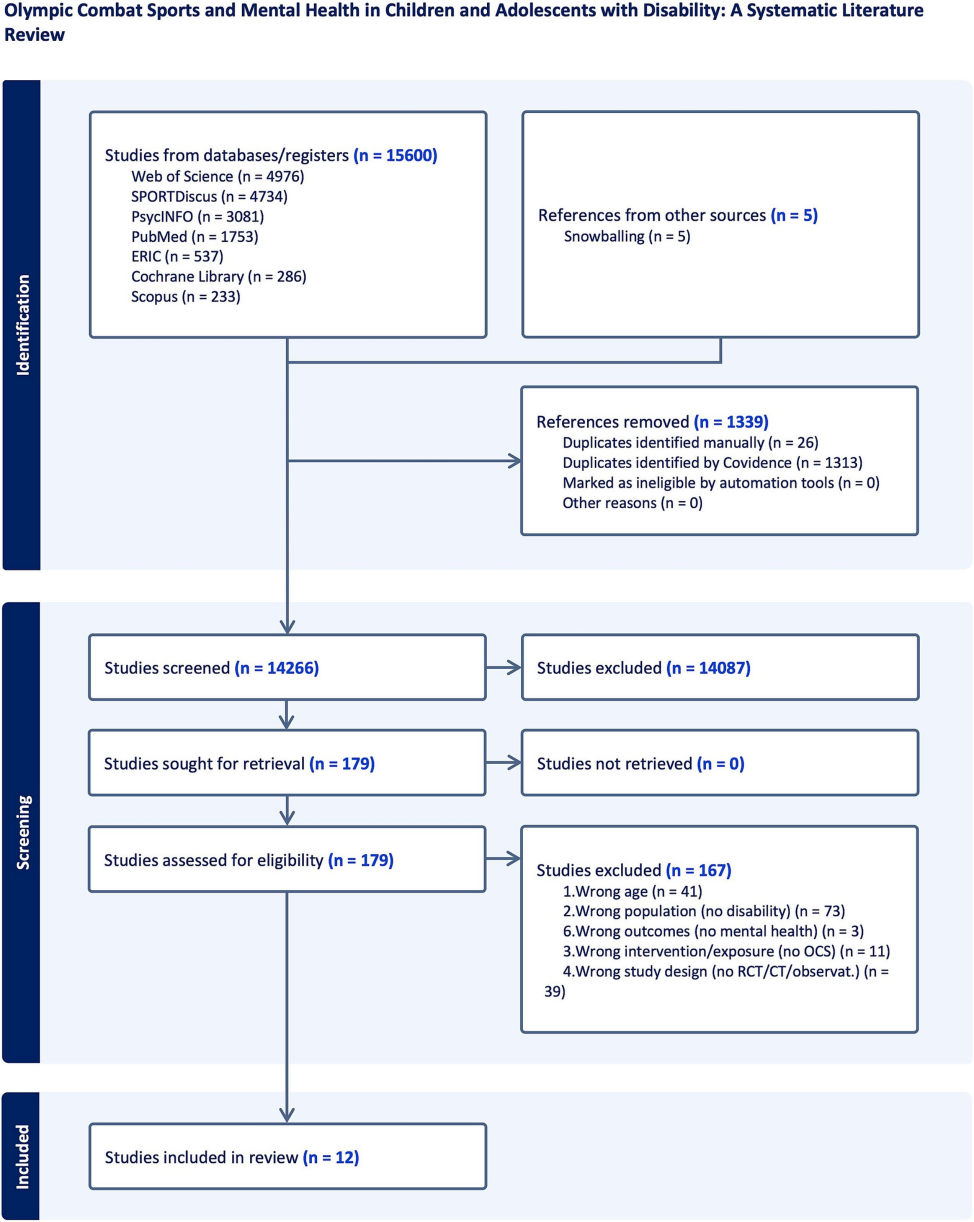
Flow diagram.

Twelve studies, seven RCTs, and five CTs from 1975 to 2022 met the inclusion criteria. Five studies focused on karate, three on judo, two on both karate and judo, one on boxing, and one on taekwondo, all with very low levels of practice. Among the 12 selected studies, only two were acute exercise studies, investigating the transient effects of a single bout of OCS activities, whereas the other 10 were chronic training interventions, investigating the effects of prolonged OCS practice. The studies spanned different regions, with six studies in Asia, four in Europe, and two in the United States. Overall, 436 children – mainly boys, aged 11.4 ± 2.8 years old – were divided into exercise or control groups in educational or training facilities. DD encompassed autism spectrum and learning disorders, whereas physical disabilities included developmental coordination disorders. Significant improvements (*p* < 0.05) in stereotypy, communication, socio-emotional and executive functions, personality, and sensory organization were reported. Furthermore, multiple studies measuring psychological (e.g., mood, self-esteem) and physical functioning (e.g., motor performance) showed improvements (*p* < 0.05), and two studies monitoring OT and CT showed immediate hormonal changes immediately after OCS training, with adolescents experiencing more significant acute decreases in CT (Figure 4).

### 3.1 Design and quality appraisal of the included studies

In our review, the seven RCTs randomly assigned people to either an intervention or control group. The five CTs, by contrast, used non-random allocation techniques. Blinded outcome judges participated in most studies. The studies reviewed had varying sample sizes but were generally large enough to have reasonable statistical power. Each study also offered specific criteria for inclusion and exclusion. However, recruiting subjects from specialty schools or clinics impairs generalization because children with developmental disabilities might dominate the homogeneous samples. Lastly, the type of control groups varied between studies (e.g., some had passive controls, some active, and others did not) (Table 1).

**Table 1:** Table of included studies.

**Author (year)**	**Design**	**OCS**	**Country**	**Number of participants**	**Group (number)**	**Disability**	**Female**	**Average age**	**Training load**	**Duration**
Bahrami et al. (2012)	RCT	Karate (specifically Kata techniques)	Iran	30	Exercise group (15) and Control group (15)	ASD	13.3%	10.5	4 times per week, 56 sessions in total	14 weeks
Bahrami et al. (2016)	RCT	Karate	Iran	30	Exercise group (15) and Control group (15)	ASD	13.3%	10.5	4 days per week, sessions gradually increased from 30 to 90 min	14 weeks
Boguszewski et al. (2013)	CT	Judo	Poland	73	Experimental group (35) and reference group (38)	ID	52.1%	11.7	Not detailed	52 weeks
Davis and Byrd (1975)	RCT	Judo	United States	16	Experimental group (8) and Control group (8)	ID (I.Q. scores: 55–80)	NI	14.5	One hour a day, three days a week	36 days in 12 weeks
Fong et al. (2012)	RCT	Taekwondo	China	62	DCD-TKD group (21), DCD-control group (23), and normal-control group (18)	DCD	NI	7.5	Weekly 1-h session, plus daily home exercises	12 weeks
Greco and De Ronzi (2020)	RCT	Karate	Italy	28	Intervention group (14) and waitlist control group (14)	ASD	14.3%	9.5	Two times per week, 45 min each session	12 weeks
Harwood-Gross et al. (2020)	CT	Judo and Karate	Israel	71	High-risk youths (31) and low-risk youths (40)	Learning disabilities	0%	16.3	<12 months (white belts) to >7 years (black belts)	Single martial arts session
Harwood-Gross et al. (2021)	CT	Judo and Karate	Israel	49	Martial arts training group (24) and control group (25)	Learning disabilities	0%	15.9	Twice a week	26 weeks
Movahedi et al. (2013)	RCT	Karate	Iran	26	Exercise group (13) and control group (13)	ASD	15.4%	10.5	1 session/day, 4 days/week	14 weeks
Palermo et al. (2006)	RCT (pilot)	Karate	Italy	16	Exercise group (8) and control group (8)	ODD	NI	9	Wa-Do-Ryu Karate program (3 sessions/week)	43 weeks
Renziehausen et al. (2022)	CT	Judo	United States	15	Exercise group (15)	ASD	NI	12	40–55 min/session x 20 sessions warm-up (5–10 min) + individual and partner-centered exercises (30–35 min) + cool down (5–10 min)	10 weeks
Tetteroo et al. (2012)	CT	Boxing	Netherlands	20	Autistic children (11) and typically developing children (9)	ASD	NI	9	Six games on the console: two training rounds and four games against the confederate.	Single experimental session

OCS, Olympic combat sports; RCT, randomized controlled trial; CT, controlled trial; DCD, developmental coordination disorder; TKD; taekwondo; ASD, autism spectrum disorder; ID, intellectual disability; ODD, oppositional defiant disorder; NI, no information.

### 3.2 Quantitative variables

To test how OCS training influences various psychological outcomes for children and young people with disabilities, the 12 included studies measured the full range of mental health-related variables outlined in Table 2, spanning three conceptual frameworks: mental illness attitudes (e.g., stereotypy, aggression), social skills (e.g., communication, social competence), and mental health literacy (e.g., self-esteem, mood, executive function, stress hormones). Table 3 displays essential statistical findings from different studies by showing outcome domains, effect directions, significance levels, and effect sizes where this information exists. Incorporating this feature aimed at improving the interpretability and transparency of results in our narrative synthesis framework. These studies mainly evaluated improvements in social skills (e.g., social–emotional functioning, communication, socialization), executive function (i.e., inhibition, processing speed, flexibility), and behavioral performance (including aggression and externalizing behaviors). They also measured physiologic effects through OT and CT testing to understand how they related to behavioral and psychological changes under the interventions. Independent samples t-tests, repeated measures ANOVAs, and linear regressions were applied to assess significant associations between OCS training and perceived psychological and cognitive benefits. These analyses offered a valuable model to explain how OCS interventions helped improve psychological well-being. The studies provided a holistic view of the variables by including group and individual data. Overall, findings showed that OCS training can effectively intervene in various psychological outcomes in children and adolescents with disabilities. This integrative approach captures the multifaceted potential of OCS training and offers insight into its therapeutic potential.

**Table 2:** Summary of mental health-related variables evaluated in the included studies.

Framework	Outcome category	Variable	Definition
Mental illness attitudes	Stereotypy	Stereotypy	Repetitive, invariant actions performed without a clear purpose or function, like hand-flapping or body-rocking (Keller et al., 2021).
Mental illness attitudes	Aggression	Externalizing behaviors	Externalizing behaviors are actions directed outwardly toward others or the environment, typically characterized by aggression, defiance, and rule-breaking (Wiggers and Paas, 2022).
Mental illness attitudes	Aggression	Oppositional behaviors	Oppositional behaviors typically refers to patterns of behavior where an individual, often a child or adolescent, displays actions that are defiant, disobedient, and hostile toward authority figures and standard norms (Ljungström et al., 2020).
Social skills	Communication	Communication deficit	It involves difficulties in developing, understanding, and using both verbal and nonverbal forms of communication (Association AS-L-H, 1993).
Social skills	Communication	Communication	The process of transmitting information through verbal and non-verbal messages. It involves a sender, a receiver, and a channel of communication (Munodawafa, 2008).
Social skills	Communication	Mimicking expressiveness	Mimicking expressiveness refers to the ability to replicate or imitate the emotional expressions or behaviors of others (Tetteroo et al., 2012).
Social skills	Social competence	Socialization	The process by which individuals learn and adapt to the norms, values, and behaviors necessary to function within a given society or social group (Lehman et al., 2004).
Social skills	Social competence	Social–emotional functioning	The skills and abilities that allow individuals to handle emotions, interact and communicate with others effectively, and form healthy relationships (Lehner et al., 2022).
Social skills	Social competence	Social dysfunction	Social dysfunction refers to various conditions within a social system that hinder its proper functioning, leading to adverse outcomes such as decreased social cohesion, increased conflict, or undermined societal stability (Lysaker et al., 2021).
Mental health literacy	Quality of life	Lifestyle	The collection of habits, behaviors, and daily practices that individuals choose or adopt, influencing their overall health and quality of life (Hautekiet et al., 2022).
Mental health literacy	Quality of life	Behavioral changes	Behavioral changes refer to modifications in an individual’s actions, responses, or behaviors due to internal or external influences. This concept is grounded in various psychological models that examine how behaviors are learned, maintained, or modified (Michie et al., 2018).
Mental health literacy	Stress hormones	Stress hormones	Cortisol is a hormone released by the adrenal cortex as part of the hypothalamic–pituitary–adrenal (HPA) axis and plays a critical role in stress regulation (Thomas and Larkin, 2020). Oxytocin, often associated with social bonding and stress reduction, can modulate cortisol levels during stress (Goetz et al., 2021).
Mental health literacy	Self-esteem	Personality	A stable and enduring patterns of thoughts, feelings, and behaviors that distinguish individuals from one another. It encompasses a broad range of traits and characteristics that manifest consistently across various situations and over time (Uher, 2017).
Mental health literacy	Self-esteem	Independence	The ability of individuals to manage their activities of daily living without assistance. This encompasses basic tasks such as bathing, dressing, feeding, and toileting (Mlinac and Feng, 2016).
Mental health literacy	Intensity	Intensity	Denotes the emotional reactivity (positive or negative) to various stimuli. Differs from “severity of [disorder],” which refers to clinically measured disorders such as depression or anxiety (Masi et al., 2021).
Mental health literacy	Mood	Mood	The amount of pleasant or unpleasant behavior observed in various situations (Amado-Boccara et al., 1993)
Mental health literacy	Adaptability	Adaptability	The ease or difficulty with which reactions to stimuli can be modified in a desired way (Hassian et al., 2022).
Mental health literacy	Executive function	Cognitive functions	Mental processes (perception, attention, memory, language) enabling us to receive, process, store, and use information from the environment (Zhang, 2019).
Mental health literacy	Executive function	Psychological functions	Higher-order processes that affect cognition, emotion, and behavior (e.g., attention, memory, executive functions), vital for interacting with the world (Poldrack et al., 2012).
Mental health literacy	Executive function	Executive function	Executive Function is a set of cognitive skills, including working memory, flexibility, and impulse control, that enable goal-directed behavior, decision-making, and self-regulation (Gilbert and Burgess, 2008).

**Table 3:** Summary of quantitative outcomes across included studies.

Author (year)	MH outcomes	Measurement tool(s)	Sample size	Effect Direction	*p*-value(s)	Effect size(s)	Timepoint(s)	Follow-up (Yes/No)
Bahrami et al. (2012)	Stereotypic behavior	GARS-2 (Stereotypy subscale)	Exercise (*n* = 15), Control (*n* = 15)	↓ (exercise group)	*p* < 0.001	d = 0.88* (approx. From t = 5.94, df = 14)	Pre, Post (14 weeks), 1-month Follow-up	Yes
Bahrami et al. (2016)	Communication deficit	GARS-2 (Communication Subscale)	Exercise (*n* = 15), Control (*n* = 15)	↓ in deficit (exercise group)	*p* < 0.001 (exercise pre-post), *p* = 0.43 (post-follow-up)	NR; Mean *Δ* = −6.18 ± 3.06	Pre, Post (14 weeks), 1-month Follow-up	Yes
Boguszewski et al. (2013)	Communication, self-confidence, assertiveness, socialization, lifestyle, independence	Parent-reported 5-point Likert survey; chi-square and t-tests	Judo group (*n* = 35), control (*n* = 38)	↑ (judo group)	*p* < 0.001 (most outcomes)	NR	Post-intervention (12 months)	No
Davis and Byrd (1975)	Personality, achievement	California Test of Personality (CTP), Wide Range Achievement Test (WRA)	Exercise (*n* = 8), Control (n = 8)	NA	NA	NA	Pre, Post (12 weeks)	No
Fong et al. (2012)	Sensory organization	Sensory Organization Test (SOT)	DCD-TKD (*n* = 21), DCD-Control (*n* = 23), Normal-Control (*n* = 18)	↑ (TKD group)	Vestibular ratio: p < 0.001; Visual ratio: *p* < 0.001; Composite SOT: *p* = 0.001	NR	Pre, Post (12 weeks)	No
Greco and De Ronzi (2020)	Social skills, Problem behaviors, Executive functioning	SSIS-RS, BRIEF	Intervention (*n* = 14), Control (*n* = 14)	↑ Social skills, ↓ Problem behaviors, ↓ executive dysfunction (intervention group)	*p* < 0.001 (all outcomes)	Social skills: *d* = 2.85; Problem behaviors: *d* = 2.64; BR: *d* = 1.36; ER: *d* = 1.63; CR: *d* = 1.54; GEF: *d* = 0.97	Pre, Post (12 weeks)	No
Harwood-Gross et al. (2020)	Oxytocin (OT), Cortisol (CT) reactivity	Salivary ELISA assays	High-risk (*n* = 31), Low-risk (*n* = 40)	↑ OT, ↓ CT (high-risk group)	OT: p < 0.001, CT: *p* < 0.01 (interaction effects)	NR	Baseline, Peak Training, Cool-down	No
Harwood-Gross et al. (2021)	Inhibition and shifting, processing speed, self-esteem, aggression	CANTAB (MTT, RVP), Rosenberg Self-Esteem Scale, Aggression Scale	Martial Arts (*n* = 20), Control (*n* = 19)	↑ Inhibition/shifting, ↑ Processing speed, ↔ Self-esteem, ↔ Aggression (martial arts group)	Inhibition: *p* = 0.003, Processing speed: *p* = 0.002, Self-esteem: *p* = 0.64, Aggression: *p* = 0.85	Inhibition: *d* = 1.02, Processing speed: *d* = 1.09, Self-esteem: *d* = 0.15, Aggression: *d* = 0.06	Pre, Post (6 months)	No
Movahedi et al. (2013)	Social interaction	GARS-2 Social Interaction Subscale	Exercise (*n* = 15), Control (*n* = 15)	↑ (exercise group)	p < 0.001	η^2^ = 0.38 (interaction), η^2^ = 0.42 (within), η^2^ = 0.15 (between)	Baseline, Post (14 weeks), Follow-up (1 month)	Yes
Palermo et al. (2006)	Intensity, Mood, Adaptability	Carey Temperament Scales	Intervention (8), Control (8)	↑ Karate group > ↓ Control	Intensity: 0.000 Mood: 0.027 Adaptability: 0.036	NR	Pre, Post (10 months)	No
Renziehausen et al. (2022)	Stress (via cortisol level)	Salivary cortisol (4 samples)	Children (*n* = 5), Adolescents (*n* = 7)	↓ (acute, adolescents only)	*p* = 0.057 (acute × age interaction)	Cohen’s *d* = 1.20 (adolescents), *d* = 0.01 (children)	Week 1 (pre/post), Week 10 (pre/post), 4 total samples	No
Tetteroo et al. (2012)	Mimicking expressiveness of movement (social skill proxy)	Observational expressiveness (custom coding scheme), Kinect-based motion capture (speed × distance)	ASD (*n* = 11), TD (*n* = 9)	↓ (ASD < TD)	Kinect: *p* = 0.008 (TD group), Obs: ns	NR	During 4 game sessions (Wii Boxing)	No

OCS, Olympic combat sports; RCT, randomized controlled trial; CT, controlled trial; DCD, developmental coordination disorder; TKD, taekwondo; ASD, autism spectrum disorder; ID, intellectual disability; ODD, oppositional defiant disorder; TD, typically developing; NI, no information; MH, mental health; GARS-2, Gilliam Autism Rating Scale, Second Edition; SSIS-RS, Social Skills Improvement System Rating Scales; BRIEF, Behavior Rating Inventory of Executive Function; BR, Behavioral Regulation Index; ER, Emotional Regulation Index; CR, Cognitive Regulation Index; GEF, Global Executive Composite Function; SOT, Sensory Organization Test; SDQ-t, Strengths and Difficulties Questionnaire for Teachers; CANTAB, Cambridge Neuropsychological Test Automated Battery; MTT, Multitasking Test; RVP, Rapid Visual Information Processing; ELISA, enzyme-linked immunosorbent assay; OT, oxytocin; CT, cortisol; WRA, Wide Range Achievement Test; CTP, California Test of Personality; η^2^, eta squared; d, Cohen’s d; Δ, mean difference; ns, not significant; ↑, increase or improvement; ↓, decrease or reduction; ↔, no change; NR, not reported.

### 3.3 OCS effects on mental health

#### 3.3.1 Mental illness attitudes

##### 3.3.1.1 Stereotypy

Bahrami et al. (2012) investigated how stereotypical attitudes affected the mental health of ASD patients. This experiment tested whether training in Kata techniques affected stereotypy and its association with mental health in 30 ASD children ages 5–16. They randomly divided participants into either an exercise group or a control group. In this exercise group, 14 weeks of Kata training four times a week resulted in a 42.54% reduction in stereotypy compared with the control group, which remained unchanged. A two-factor mixed-model ANOVA revealed a significant group × time interaction (*p* < 0.001), and post-hoc paired *t*-tests showed a significant reduction in stereotypy scores for the exercise group from baseline to post-intervention (*t*(14) = 5.94, *p* < 0.001), with no significant changes observed in the control group (*t*(14) = 1.10, *p* = 0.29). Notably, that decrease was maintained for 30 days after the intervention.

##### 3.3.1.2 Aggression

Harwood-Gross et al. (2021) investigated whether a six-month martial arts training course reduces aggression in at-risk teenage boys. While there was no meaningful difference in self-reported aggression levels between participants who underwent martial arts training and a control group who received regular physical education, early hormone changes–oxytocin sensitivity–emerged as a reliable predictor of aggression levels. Those who displayed more significant oxytocin levels in the initial training periods were more aggressive after the intervention.

#### 3.3.2 Social skills

##### 3.3.2.1 Communication

Four studies investigated the impact of OCS training on the communication skills and the mental health of individuals with developmental disabilities, including ASD and intellectual disabilities.

Movahedi et al. (2013) evaluated the impact of Kata techniques training on verbal abilities and mental health in 30 ASD children between 5 and 16 years. Participants were randomly assigned to an exercise group, where participants were instructed in Kata training four times a week for 14 weeks, and a control group. Significant improvements in social dysfunction were observed, contributing to decreased stress and anxiety. A repeated-measures ANOVA revealed a significant group × time interaction (*p* < 0.001), with the exercise group showing a 40.32% reduction in social dysfunction scores from baseline to post-intervention [*t*(12) = 6.17, *p* < 0.001], while the control group exhibited no significant changes [*t*(12) = 0.62, *p* = 0.55].

Boguszewski et al. (2013) examined judo classes to determine how they affected the language skills and mental health of intellectually disabled children. Their participants were 73 children, 35 of whom attended an exercise group conducting judo lessons and the remainder in a control group. The exercise group reported impressive improvements in self-confidence, assertiveness, and communication skills. Children in the exercise group showed a statistically significant increase in socialization scores compared to the control group (*p* < 0.001), with 80% of parents reporting marked progress in their children’s ability to communicate with their environment. Improvements in lifestyle scores (*p* < 0.001) were also noted, reflecting enhanced daily functioning and adherence to social norms. Such increased communication was correlated with improved mental health: higher confidence and social contact helped reduce anxiety and depression.

Tetteroo et al. (2012) examined expressiveness and mimicking behaviors in children with ASD using a gamification approach. In 20 children (11 with ASD and 9 typically developing), the experiment used the Wii boxing game to quantify expressive behavior. Children with ASD demonstrated challenges in mimicking the expressive movements of their peers, reflecting social communication challenges. A significant difference in expressiveness scores was observed between typically developing children and children with ASD, as measured by the Kinect Expressiveness metric (*p* = 0.008), with typically developing children scoring higher. The control group’s behavior was significantly influenced by the confederate’s level of expressiveness (*p* < 0.01), while the ASD group showed no such influence. These limitations went together with increased stress and frustration.

Lastly, Bahrami et al. (2016) studied the social and communicative improvement of children with developmental disabilities using karate. The trial, conducted over 14 weeks, included karate sessions to improve communication deficits. Researchers found significant gains in social engagement, instruction, and communication in general. A significant reduction in communication deficits for the exercise group [mean difference = −6.18, *t*(14) = 6.70, *p* < 0.001] from baseline to post-intervention, whereas no significant change was observed in the control group [*t*(14) = 0.72, *p* = 0.49]. These developments were closely linked to more significant mental health.

##### 3.3.2.2 Social competence

Three studies investigated the impact of martial arts training on social competence and mental health in individuals with developmental disabilities, including ASD and intellectual disabilities.

Greco and De Ronzi (2020) tested whether a 12-week karate program helped children with ASD develop their social–emotional and executive skills. The researchers randomly assigned 28 children aged 8–11 to an intervention or waitlist control group. The intervention group was taught Kata techniques twice a week. Test results demonstrated significant increases in social competence, including communication, cooperation, and engagement, as well as decreased aggression and hyperactivity. A significant increase in social skills scores for the intervention group [*t*(13) = 10.69, *p* < 0.001, Cohen’s *d* = 2.85], while problem behaviors decreased significantly [*t*(13) = −9.77, *p* < 0.001, Cohen’s *d* = 2.64]. Such social skills correlated with other outcome domains of mental health.

Boguszewski et al. (2013) tested how judo training enhanced social skills and mental health in children with intellectual disabilities. The experiment involved 73 children, of whom 35 attended judo lessons. Judo practitioners showed remarkable improvements in adapting to their surroundings, socializing and conforming, and developing greater emotional regulation. Statistically significant differences were observed between the judo and control groups in key areas of communication and socialization (mean increase of 3.0 points on a 5-point scale, *p* < 0.001). Furthermore, self-confidence and assertiveness improved significantly in 69% of participants, while 80% of parents reported enhanced communication abilities in their children. Such gains were statistically significant compared with the control group and related to higher self-esteem, assertiveness, and mental health.

Movahedi et al. (2013) examined the influence of 14 weeks of Kata training on social dysfunction and mental health in 30 children with ASD. The study found notable improvements in social skills through routine karate classes and decreased negative traits, including stereotypy and aggression, in the exercise group. The exercise group showed a 40.32% reduction in social dysfunction scores from baseline to post-intervention [*t*(12) = 6.17, *p* < 0.001], while no significant changes were observed in the control group [*t*(12) = 0.62, *p* = 0.55]. These improvements were maintained at a one-month follow-up, with no significant differences in scores compared to post-intervention [*t*(12) = −1.65, *p* = 0.13]. Such social benefits were highly correlated with improved mental health, with participants having less anxiety and greater self-control.

#### 3.3.3 Mental health literacy

##### 3.3.3.1 Quality of life

Boguszewski et al. (2013) assessed how judo training affected the mental health and quality of life of 73 children with intellectual disabilities (median age = 11.7 years, SD = 2.6). Thirty-five underwent a judo intervention alongside their standard therapy, while 38 served as controls. Although the frequency and duration of the judo sessions were not stated in the paper, results showed that children in the judo group performed significantly better (*p* < 0.001) in multiple areas, such as communication with the environment, lifestyle adaptation (working at home and in the community) and socialization (social conformity, emotional regulation). These children scored over 3 points on the five-point scale for communication, lifestyle, and socialization skills and about 2.5 points on self-care skills. As many as 80 percent of parents noticed improved communication; on average, 69% noticed greater self-confidence and assertiveness in the judo group. In addition, the total level of rehabilitation was assessed at 4.71 (out of 5) points for the judo and 3.55 (*p* = 0.001) points for the control groups.

##### 3.3.3.2 Stress hormone

Two studies focused on the relationship between physiological (stress hormones, CT, and OT) and behavioral mental health outcomes in the context of martial arts training among children with ASD and high-risk youths.

Renziehausen et al. (2022) tested the feasibility and early impact of a 10-week community-based judo program on cortisol levels in children with ASD. There were 17 aged 6 to 17 split into children (8–12 years) and teenagers (13–17 years). Twelve of these (71%) submitted all four required salivary cortisol samples (before and after both the first and last session). Although no chronic cortisol change was detected during the 10-week study, acute drops in cortisol were seen immediately after judo practice. This trend was even more pronounced in adolescents, where the effect size (Cohen’s *d* = 1.2) was more significant than for children (Cohen’s *d* = 0.01).

Harwood-Gross et al. (2020) looked at hormonal responses – specifically OT and CT – in a martial arts boot camp with high-risk (*n* = 31) and low-risk (*n* = 40) adolescents. The high-risk participants had significantly lower OT scores at baseline than the low-risk participants (*p* < 0.01), but baseline CT scores were broadly similar between groups. While both groups experienced OT elevations throughout the session, only the high-risk youths maintained their higher OT levels post-session, leaving no significant group difference after training. In the case of CT, young adults at low risk showed significant CT reactivity from baseline to peak training (*p* = 0.01) and sustained this after post-training (*p* < 0.001), while those at high risk showed no CT reactivity.

##### 3.3.3.3 Self-esteem

Harwood-Gross et al. (2021) also tested whether their martial arts course reduced the self-esteem of at-risk teenage boys. Although the intervention significantly improved inhibition, shifting, and processing speeds, the research showed no significant differences in self-esteem between the martial arts group and a control group receiving traditional physical education. However, one key finding emerged from hormonal analyses: the more people were cortisol-reactive early in the intervention, the more they benefited from training their self-esteem.

##### 3.3.3.4 Intensity

Palermo et al. (2006) assessed the impact of a 10-month structured karate program on the behavioral symptoms of children with ODD. In this context, “intensity” refers to how emotionally responsive (good and bad) someone is, not to how severe a disorder or symptom might be. Three times a week, children exposed to karate sessions reduced overall reactivity much more than children without such intervention. Toward the end of the program, these children appeared to be much less belligerent and impulsive, demonstrating better self-control. This decreased emotional reactivity was related to fewer disruptive episodes and greater flexibility and mood control at home and school. Significantly, onset levels of intensity correlated with post-intervention outcomes; those participants who were less reactive had lower levels of aggression and defiance over the long term.

##### 3.3.3.5 Mood

Palermo et al. (2006) also examined the effects of their karate program on children with ODD in temperament areas, including intensity, flexibility, and mood control. The mood regulators assessed the positive and negative behavior ratio across various situations. The karate group, who trained three times a week and joined classes with typically maturing colleagues, showed significant mood regulation changes by the end of the intervention. The control group that did not participate in the program made no such gains.

##### 3.3.3.6 Adaptability

Palermo et al. (2006) also examined the effects of their karate intervention on ODD children and focused heavily on adaptability as a temperamental trait. After the intervention, children in the karate group were much more adaptable –flexible enough to alter reactions and behavior in response to changing conditions than control groups. These improvements translated into increased adaptive scores at the program’s endpoint (T1), even when comparable baseline (T0) measures were in play.

##### 3.3.3.7 Executive function

Of the studies included, one study specifically explored the potential impact of OCS training on executive functioning in children with ASD. Greco and De Ronzi (2020) conducted a 12-week Kata-focused karate intervention for 8–11-year-old ASD children. Compared to a waitlist control group, the karate group showed statistically significant gains across the major executive function domains, including behavior inhibition, working memory, and cognitive flexibility. The intervention group also reported lower levels of emotional and behavioral challenges (aggression, hyperactivity, anxiety) after the intervention. Most significantly, the parents of children in the karate condition were quite happy with the training style and eager to continue after the study.

### 3.4 Moderating factors

#### 3.4.1 Age

One included study investigated the association between age and outcomes, showing differential effects over time. Renziehausen et al. (2022) tested the feasibility and early impact of a judo community intervention on cortisol levels in children with autism spectrum disorder (ASD). The subjects ranged in age from 8 to 17 years, broken up into children (8–12 years) and adolescents (13–17 years). Results showed that cortisol was lowered significantly at the most critical times in adolescents aged 13–17, indicating an age-specific difference in physiological stress response to the intervention. A significant main effect for age was noted (*p* = 0.009), with adolescents exhibiting a higher reduction in acute cortisol levels (Cohen’s *d* = 0.979) compared to children (Cohen’s *d* = 0.055). Overall, this paper reveals a complex relationship between age and intervention outcomes.

#### 3.4.2 Family factors

One study sought how a structured, 10-month karate program might have influenced behavioral domains—intensity, flexibility, and mood regulation of children with ODD and externalizing behavior (Palermo et al., 2006). Although the authors did not discuss the factors affecting familial interactions, they concluded that continuous caregiver engagement—including parents taking the children to each session—strongly contributed to the adequacy reduction. Karate-trained students improved significantly across all temperament ranges because the martial arts intervention helped enhance emotions. Furthermore, the authors said that it was consistent with many treatment programs emphasizing “effective parenting strategies,” suggesting that consistent discipline and parental engagement can significantly enhance children’s grow.

## 4 Discussion

This systematic review indicates that participation in OCS–judo, karate, boxing, taekwondo, and mixed approaches, which incorporate multiple components such as physical activity, psychological education, and social interaction training–can positively change the mental health of children and adolescents with disabilities. Even though the reviewed studies were somewhat varied in combat sports, intervention frequency and duration, study design, and disability groups addressed, their convergent positive findings lent credence to the possibility that OCS-based programs might offer complementary interventions for children and adolescents with disabilities. Specifically, this review showed consistent advancements in verbal communication skills, social interaction abilities, emotional regulation, and stress management techniques. Recent research confirms that structured physical activities that promote consistency and cooperation while creating a shared purpose help support socio-emotional and psychological growth among children and adolescents with developmental, intellectual, and sensory impairments (Smith and Sparkes, 2019; Shen et al., 2024; Puce et al., 2023; van Lindert et al., 2023). However, for all these early clues, some subtle observations need to be carefully considered, and these findings deserve discussion of both the clinical and methodological contexts within which they emerged.

The overall theme emerging from these studies is that OCS-mediated interventions can help to build social and communicative skills in children and adolescents with disabilities, especially those with ASD or mild cognitive disability. In some included studies, karate-trained participants experienced statistically significant improvements in communication, social impairment, self-expression, and social involvement (Movahedi et al., 2013; Harwood-Gross et al., 2021; Bahrami et al., 2016; Greco and De Ronzi, 2020). Specifically, ASD children typically show limited social reciprocity, deficits in nonverbal communication, and poor abilities to interpret other people’s emotional signals (Lord et al., 2018). Thus, the class atmosphere of a martial arts course, with repetitive forms or moves that are practiced in groups or pairs, may help children become socially comfortable (Giardullo et al., 2024; Calinog et al., 2021). Moreover, Bahrami et al. (2012) and Bahrami et al. (2016) observed that practicing kata moves reinforced stable motor patterns and offered repeated social practice and guided interaction. Along with these increases in social competence, participants exhibited reduced stereotyping and anxiety, indicating that strengthening adaptive social behaviors can also help reduce stress in children and adolescents vulnerable to sensory overexposure or communicative frustration. Thus, the repetitious, ritualistic nature of martial arts and the help of supportive instructors could account for the success of these interventions in groups with ASD or other communication impairments.

Researchers working in the broad field of psychosocial well-being have also demonstrated that OCS can strengthen children’s resilience, confidence, and intercultural skills. Previous studies (Davis and Byrd, 1975; Boguszewski et al., 2013) included in this review labeled participants as “mentally retarded” or “educable mentally retarded” (I.Q.: 55–80), which reflected the language then in use. In practice, those terms are generally replaced by “intellectual disability,” which better reflects people’s varying abilities and the requirement for person-centered terminology. This change in definition coincides with a more significant shift in research and public awareness: just as the knowledge of intellectual and developmental disabilities increased, so too did awareness of the necessity of inclusive, respectful language. As such, the nomenclature shift reflects a linguistic aesthetic and a qualitative shift in how we conceptualize, research, and treat people with intellectual disabilities. Boguszewski et al. (2013) highlighted that intellectually disabled children undergoing a judo training program performed well on socialization measures, with parents reporting that children became confident and adjusted to the everyday world at school or home. The discipline of OCS training–in which learners are taught to respect instructors and adhere to firm codes of conduct–might be the key to these goals, as learners are regularly expected to exercise self-control, observe group standards, and act responsibly toward their trainers (Hetman and Tymchyk, 2023). Repetitions of these structured habits can be translated into more constructive and controlled action outside the martial arts environment.

One intriguing aspect of the studies included the impact of stress-induced physiological processes – in this case, CT and OT. Renziehausen et al. (2022) reported that while judo training administered for 10 weeks did not cause long-term changes in cortisol secretion in children with ASD, there were immediate acute reductions immediately following training, particularly in adolescents. The production of cortisol is affected by biological maturation since pubertal development stages differ among children and adolescents (Knutsson et al., 1997; Natsuaki et al., 2009). The observed age-dependent decrease in cortisol levels after OCS training might be partially explained by a different responsiveness of the hypothalamic–pituitary–adrenal (HPA) axis during different stages of development in children and adolescents (Filetti et al., 2024). Researchers must examine external stress factors and the internal developmental stage when analyzing physiological measures like cortisol levels. And the sudden drops in cortisol mean a short-term break from stress – which may be helpful in those who find themselves physiologically roused by a change in the environment or by social stimulation. Incorporated into a broader behavioral program, these periodic acute drops can help to produce a more adaptive stress response over time and manage anxiety in challenging conditions (Ciaccioni et al., 2024). Moreover, Harwood-Gross et al. (2020) found that at-risk children who engaged in martial arts training had higher OT levels post-training, putting them on an equal footing with the low-risk group. Since OT is associated with prosocial behavior and attachment (Singer et al., 2008), elevated hormone levels could account for at least some social motivation benefits observed in martial arts interventions. OCS’s ordered, cooperative, and reassuring behaviors (bowing, pair drilling, group challenges) can enhance social trust and states of positive arousal (Bojanić et al., 2019). However, more studies are needed to explore whether OT or CT changes were transient or if more sustained changes could follow exposure to these interventions in the physiology of stress and social engagement.

Researchers have also examined whether OCS training can change at-risk youth’s aggression, emotional regulation, and self-confidence. While parents and clinicians may be concerned that OCS, by nature of their aggressive nature, exacerbates aggression, the studies presented here do not show increased aggression consistently. Instead, there is a raft of evidence that the rule-based organization of OCS might allow disruptive children and adolescents to focus their energies on practice, becoming able to harness sudden flare-ups. For instance, Palermo et al. (2006) found that a 10-month karate course significantly reduced the intensity of emotional reactivity, improved flexibility, and decreased mood dysregulation in children who display oppositional defiant behavior. Participants who had completed the training often seemed more peaceful and exhibited less defiant behavior at home and school. This point highlights that OCS instruction, while focused on battle, fundamentally differs from physical aggression unbridled. This heavy-handed emphasis on respect, in-depth rule-following, and sparring under tight supervision may improve self-regulation among impulsive adolescents (Babinski et al., 2023; O’Connor and Colder, 2015). Moreover, by laying down principles about the improper and proper use of force, martial arts might help children learn to be more sensitive to their feelings, perhaps avoiding inappropriate violence in haphazard everyday situations.

Aside from communication, socialization, and aggression management, specific interventions have also provided insight into participants’ self-esteem and autonomy. Children with disabilities experience stigma, fewer peer connections, and less accessible recreational experiences, which undermine their confidence and independence (Fine and Asch, 1988; Casas, 2007). In some studies, the continuous repetition of techniques associated with different belt ranks and the gradual mastery of new moves seemed to increase participants’ feelings of accomplishment and self-esteem. While not all studies observed a net increase in self-esteem following the intervention, several reports did indicate that subgroups of adolescents, particularly those who showed early physiological stress reactivity, experienced significant increases in self-esteem and self-regulation (Harwood-Gross et al., 2021). This subtlety suggests that individuals with higher stress levels may be most attracted to a structured and vigorous exercise, such as judo or karate since it provides both an outlet for past-life stress and a secure environment to moderate emotional response. By piling up modest training wins, these individuals can internalize notions of competence and mastery that will translate, over time, into wider enhancements in self-confidence.

Our review examined only OCS recognized by the IOC because of their standardized rules and global accessibility while noting that non-OCS disciplines like Capoeira might also be necessary. Modern research recognizes Capoeira, an Afro-Brazilian martial art blending physical movement with music and ritual, as a “holistic movement practice” that benefits physical health and enhances cognitive abilities alongside emotional and cultural growth. New research has revealed Capoeira’s distinct advantages in child development. One systematic review found that Capoeira engages multiple brain regions involved in motor planning, executive control, and emotional processing, including the premotor cortex, supplementary motor area, and insula (Monteiro-Junior et al., 2025). This review indicates that Capoeira requires and improves high-level cognitive abilities through its dynamic movement patterns involving interaction. Another randomized controlled study revealed that children aged 8–12 who practiced Capoeira for 4 months showed substantial improvements in executive functions when they attended classes more than 70% of the time (Fernandes et al., 2022). Children aged 8 and 12 who attended Capoeira classes for 4 months showed considerable improvements in executive function, especially when participating in over 70% of their classes. This study found improvements in working memory, inhibitory control, and motor coordination, which displayed a clear dose–response relationship. Capoeira’s cooperative and expressive structure enabled children to boost their self-esteem and develop a stronger sense of engagement and cultural connection. We excluded Capoeira from our review because it does not have IOC recognition. However, these results demonstrate how movement practices outside traditional OCS classifications can produce similar or additional benefits. Future studies need to explore the relationship between culturally embedded, cognitively complex interventions and the structured environments common in Olympic sports to understand their impact on children and adolescents with disabilities’ mental health and social development.

## 5 Limitations

Simultaneously, methodological and contextual problems arise from this systematic review that calls for caution in interpreting the evidence. In the first instance, the studies were so different in design, time-to-result, outcome criteria, and disabilities addressed that common conclusions were difficult to derive. Most studies were randomized controlled trials, but some were non-randomized controlled trials; sample sizes were often small, sometimes with a small statistical power to allow meaningful differences. Moreover, control populations in such studies were a mixture of other physical activity or passive waiting-list controls that can misrepresent or overstate the effect size of the interventions. This review intentionally omitted observational studies to enable more decisive causal conclusions. This exclusion limits the generalizability of findings because it prevents from capturing information from observational research, which provides broad real-world contexts, especially within underrepresented or diverse disability populations. Subsequent reviews should consider incorporating these studies to achieve a more complete perspective. The screening process yielded numerous observational studies, which were ultimately excluded according to our established eligibility standards. Despite being excluded from the final synthesis, these studies provide meaningful context and demonstrate the necessity for upcoming reviews that encompass a broader range of study designs. Likewise, we restricted our inclusion criteria to OCS, which the IOC officially recognizes, to achieve definitional clarity and methodological clarity. The definition of OCS used in this review adheres to the sports recognized by the IOC during the study selection period. These classifications evolve throughout different Olympic cycles. The Olympic program included karate for Tokyo 2020, following its inclusion in the Buenos Aires 2018 Youth Olympic Games, but decided against its inclusion for Paris 2024. Karate was shortlisted for the 2028 Los Angeles Olympics. Still, it was not selected, while the World Karate Federation (WKF) is working with the Brisbane 2032 organizing committee to allow its inclusion within the Games. Our review methodology shows a temporal restriction because it does not incorporate non-IOC sports such as Brazilian jiu-jitsu and capoeira. Future research projects should incorporate more combat sports to fully understand their varied impacts on young people’s mental health. Nevertheless, using standardized training protocols, international regulations, and formal recognition of adapted physical activity enables researchers to compare findings across different studies. Our choice to exclude Brazilian jiu-jitsu, capoeira, and mixed martial arts from our study because they do not meet OCS criteria led to their exclusion despite their promising mental health benefits (Bueno et al., 2023; Fernandes et al., 2022; Phung and Goldberg, 2021), which warrants future systematic reviews to expand their inclusion criteria to assess all combat-based interventions for youth with disabilities. This selection process produces sampling bias and constrains our synthesis scope. Subsequent studies should investigate how non-OCS perform comparatively in this population. Whether the unique attributes of OCS (the focus on discipline, ritual, or directed body contact) are better than similar structured activities such as swimming or dancing classes is not clear. The interventions were sometimes also short-term, involving single experimentation, so longer-term effects on coping ability, psychosocial functioning, or biomarkers of stress control were not possible to measure. We will need more long-term studies with more significant numbers and better designs to investigate whether these temporary advantages translate into long-term gains in mental health, social engagement, or academic performance. Furthermore, the diversity in outcome domains, measurement tools across studies, and inconsistent statistical reporting methods prevented us from performing a formal meta-analysis. Thus, the heterogeneity, the limited number of studies, and inconsistent effect size reporting prevented us from performing meaningful quantitative synthesis.

Furthermore, with only minimal data about compliance, injuries, or adverse events, we do not know what the issues involving disabled children and adolescents could be in physical contact sports. It is conceivable that a few families or participants would end these programs if they got hurt or if the child was unable to handle the physical and social rigors of training. This research would have benefited from better reporting requirements, including details about dropout rates, safety events, and program ‘feasibility.’ Such transparency would enable teachers, clinicians, and parents to decide whether these interventions suit their children and adolescents. Furthermore, a deeper understanding of the psychosocial environment created by coaches and instructors is critical. Expert instructors training in adaptive physical education could modify the martial arts experience to suit the individual student’s capabilities and mediate results. Unstructured or insensitive instruction, on the other hand, could harm at-risk children, which is why we want trained instructors who know disability limitations and communication styles.

Moreover, instructors’ prior expectations about student potential might have unknowingly affected how OCS interventions were delivered and their subsequent results (Rosenthal, 2010). Participants receive reduced challenge and engagement when instructors underestimate their potential, restricting their motor development and psychological growth. Participants may exhibit increased confidence and participation when instructors maintain high expectations. Involving instructors in outcome assessment can introduce expectation or confirmation bias in non-blinded trials, which might lead to overestimated intervention effectiveness (Jussim, 2012). The motivational climate created by instructional styles such as inclusive support or rigid performance metrics indirectly affects participants’ emotional states and mental health outcomes (Reinboth and Duda, 2006). The lack of consistent reporting or consideration of instructional dynamics across studies might affect the interpretation of observed benefits. Subsequent research and evaluations must analyze how the pedagogical approach and instructor traits influence the success of OCS programs for youth with disabilities.

On the way forward, researchers might build on these observations in various ways. An initial option is to prolong interventions and conduct follow-up checks to help explain whether temporary changes in behavior and social functioning translate into a sustained benefit. An eye on children’s behavior months or years after intervention might allow us to understand whether repeated OCS sessions build new coping skills, foster stable social relationships, or help them avoid falling back into dysfunctional behavior. Researchers might also use targeted subgroup analyses based on age, sex, disability severity, or baseline stress physiology to determine which children are best served by OCS and which need extra care or alternative treatment. However, most of the included studies failed to report participants’ sex or presented markedly unbalanced sex distributions, often with far fewer female participants. An uneven distribution of participants by sex reduces how broadly findings apply and hinders insights into girls’ reactions to OCS-based treatments. Additionally, the developmental stages of participants were rarely considered or reported in detail. The absence of consideration for pubertal maturation introduces bias when interpreting cortisol and psychosocial outcomes because puberty triggers hormonal and psychological changes that affect stress response, mood regulation, and social behavior (Buchanan et al., 1992; Foilb et al., 2011). Researchers must report and control these factors in future studies to improve interpretation accuracy and enable valid subgroup comparisons. Qualitative data (interviews with children, parents, and teachers) would supplement existing research and shed new light on how martial arts can rewire young people’s identity, compassion, and autonomy. One key direction would be systematic research into how family participation contributes to treatment outcomes. Some, such as Palermo et al. (2006), pointed to the importance of parental support and involvement in children’s development. Families and OCS instructors could collaborate to deliver complex outcomes if parents continue to enforce discipline and practice methods at home or if they go to classes themselves to model good behavior. Studies that regularly incorporate parent coaching, family goal setting, or home practice would help to unravel the relationship between how much of an intervention’s success is down to the physical and social aspects of the sport and complementarity from a structured home.

We used a SWiM approach to accommodate the limited sample (*n* = 12) and methodological diversity. Although we attempted to adhere to the SWiM guidelines, our success in implementing elements (e.g., consistent effect-size calculations, formal heterogeneity measures, comprehensive certainty-of-evidence tests) was limited by the heterogeneity of the data reported and outcome measures. Furthermore, we utilized CFIR to improve our understanding of intervention implementation contexts but recognize that it cannot replace thorough statistical analysis. We aim to address limitations by providing Table 3, which includes effect size estimates and structured statistical outcomes to enhance interpretive precision within the narrative synthesis framework. For example, many experiments did not quantify effect sizes or standard scales, which makes transformations or group estimates difficult. Furthermore, the lack of homogeneity in studies kept more vigorous subgroup studies or quantitative explorations of heterogeneity away. We also point out that our original protocol was mainly concerned with emotional, behavioral, and social effects. However, while screening and scouring the literature, we came across a series of papers measuring cognitive functions–particularly executive functions–that are profoundly relevant to self-regulation and, consequently, mental health in children and young people with disabilities. While broadening our scope to include these cognitive effects further widened the dataset’s heterogeneity, it gave us a broader perspective on how OCS interventions might affect general psychosocial functioning. However, the interaction between cognition and emotion, the small sample size, and the non-randomized study designs raise questions about how future studies could be conducted using standardized metrics and better design. Moreover, in recognizing these limitations, we highlight both the utility and the difficulties of a narrative synthesis approach in the face of a diverse evidence base.

## 6 Conclusion

In conclusion, this systematic review supports the hypothesis that OCS interventions can benefit the mental health of children and adolescents with disabilities by strengthening communication, social skills, emotional regulation, and physiological stress responses. The results are broadly positive but require careful interpretation given the methodological variability, small sample size, and intervention protocols found across trials. Such programs should continue to be designed from a whole-person perspective, considering the integration of skill acquisition, routines, responsive instruction, and parental/carer involvement. With more substantial, significant, and longer studies, researchers, educators, and clinicians can clarify the full therapeutic promise of OCS and create research-based interventions that promote psychosocial development in children and adolescents with various disabilities. Coupling these formal physical practices with broader support networks might significantly increase access for young people with disabilities to participate in inclusive sports, build confidence, mitigate stress, and live healthier, more fulfilling lives. To support these broader goals of accessibility and inclusion, several transnational initiatives (van Lindert et al., 2023) have aimed to increase opportunities and cooperation among stakeholders to promote adapted combat sports for youth with disabilities. This framework also envisaged that the Paralympic Games and the Special Olympics include more combat sports in their programs.

## Data Availability

Publicly available datasets were analyzed in this study. This data can be found at: https://doi.org/10.5281/zenodo.14026776.
